# Role of astrocytes in obesity-induced hypertension

**DOI:** 10.3389/fnins.2026.1992730

**Published:** 2026-10-21

**Authors:** Diani Nanayakkara, Claudia Pantoja Negron, Madhan Subramanian

**Affiliations:** 1 Molecular Neurophysiology Lab, Oklahoma State University (OSU), Stillwater, OK, United States; 2 Department of Physiological Sciences, College of Veterinary Medicine, Oklahoma State University (OSU), Stillwater, OK, United States

**Keywords:** astrocytes, autonomic regulation, EAAT2, glutamate, neurogenic hypertension, neuroinflammation, obesity-induced hypertension, sympathetic nervous system

## Abstract

Although obesity is a significant contributor to hypertension and cardiovascular disease, the mechanisms that connect excess adiposity to persistently elevated blood pressure extend far beyond peripheral metabolic and vascular changes. An increasing body of evidence suggests that a substantial component of this puzzle is disrupted autonomic regulation, specifically persistent activation of sympathetic pathways. This review explores the emerging role astrocytes play in this process. By controlling extracellular glutamate, ion balance, inflammatory signaling, energy metabolism, blood brain barrier integrity and neurovascular coupling, astrocytes normally contribute to the maintenance of neuronal and vascular homeostasis. However, exposure to excess nutrients, circulating fatty acids, adipokines and inflammatory mediators can interfere with these processes and cause astrocytes to become reactive. This change may increase oxidative and inflammatory signaling, hinder glutamate clearance, and disrupt the astrocyte-neuron communication in the cardiovascular regulatory centers of the brainstem and hypothalamus. The disturbance of excitatory amino acid transporter mediated glutamate homeostasis is specially concerning because it may intensify excitatory drive in autonomic circuits and sustain sympathoexcitation. In the context of obesity-associated hypertension, this review interconnects recent data to link metabolic dysfunction, neuroinflammation, central autonomic signaling, and astrocyte dysfunction. It also considers new therapeutic approaches targeted at restoring astrocytic balance. Targeting astrocyte-dependent pathways may provide a valuable supplementary strategy to address the neurogenic aspect of obesity-induced hypertension, even though several mechanistic questions remain unanswered.

## 1 Introduction

Obesity is now understood as a chronic, relapsing disease rather than simply a matter of excess weight, arising from the interplay of biological, behavioral and environmental factors (Bray et al., 2017). In adults, body mass index (BMI), calculated as weight in kilograms divided by height in meters squared (kg/m^2^), is commonly used to categorize body weight relative to height. Overweight is defined as a BMI of 25.0–29.9 kg/m^2^, whereas obesity is defined as a BMI ≥ 30.0 kg/m^2^ and is further categorized as Class I (30.0–34.9 kg/m^2^), Class II (35.0–39.9 kg/m^2^), and Class III (≥ 40.0 kg/m^2^) Thus, overweight and obesity represent distinct BMI categories, while elevated BMI is a broader term referring to BMI values above the healthy range. Although BMI is widely used as a practical population-level measure, it is an indirect measure of adiposity and does not directly characterize body fat distribution (Nuttall, 2015; Tandar et al., 2026).

Its global prevalence has climbed sharply over the past several decades, driven largely by shifting dietary habits, declining physical activity, and changes in food environments and socioeconomic conditions (Blüher, 2019; Phelps et al., 2024). The spread of processed, high-energy foods and more sedentary lifestyles brought about by globalization and industrialized food systems have accelerated this increase in both developed and developing countries (Popkin et al., 2012). By 2022, an estimated 2.5 billion adults worldwide were classified as overweight and more than 890 millions of them were living with obesity (Phelps et al., 2024). The health toll of this trend is difficult to overstate. Elevated BMI remains one of the leading contributors to noncommunicable diseases (NCDs) worldwide (Dai et al., 2020; Zhou et al., 2024) and in 2021 alone, it was linked to approximately 3.7 million deaths, primarily through cardiovascular disease, diabetes, cancer, neurological disorders, chronic respiratory illness, and gastrointestinal conditions (Figure 1) (Brauer et al., 2024). The economic burden associated with the obesity epidemic is substantial and is projected to increase considerably if current trend persists (Okunogbe et al., 2022). The financial cost is just as striking; global obesity-related expenses are projected to approach US$3 trillion a year by 2030 and could exceed US$18 trillion by 2060, once healthcare spending, lost productivity, and other societal costs are factored in (Okunogbe et al., 2022).

**Figure 1:**
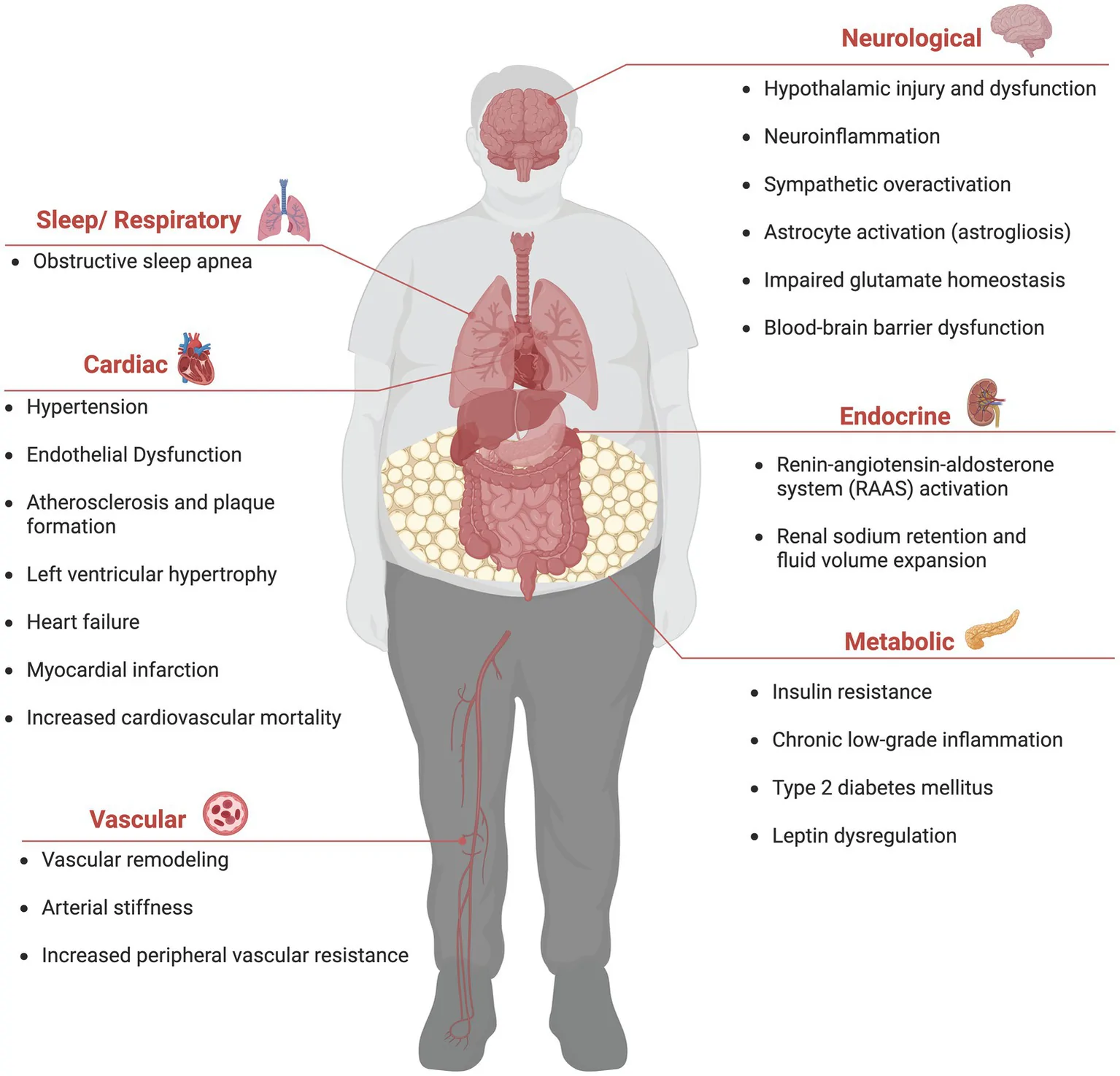
Multisystemic consequences of obesity. Obesity induces widespread pathological changes affecting multiple organ systems. Major consequences include metabolic dysfunction (insulin resistance, leptin dysregulation, and chronic inflammation), cardiovascular disease (hypertension, endothelial dysfunction, and heart failure), vascular remodeling, neuroinflammation and sympathetic overactivation, RAAS activation, and respiratory disorders such as obstructive sleep apnea. Together, these alterations contribute to increased cardiovascular risk and the development of obesity-associated hypertension.

Cardiovascular disease (CVD) is among the most well-documented consequences of obesity, which acts as an independent risk factor for its onset and progression (Powell-Wiley et al., 2021). A series of pathophysiological alterations including dyslipidemia, insulin resistance, hypertension, and chronic inflammation are triggered by excessive adiposity and collectively worsen vascular function and hasten atherosclerosis (Powell-Wiley et al., 2021). Specifically, visceral fat acts as an active endocrine organ, releasing pro-inflammatory cytokines and adipokines that harm the endothelium and encourage the development of plaque (Liberale et al., 2017). Additionally, obesity increases the risk of heart failure by causing adverse cardiac remodeling such as left ventricular hypertrophy which is exacerbated by associated conditions like type 2 diabetes and obstructive sleep apnea (Figure 1). Taken together, epidemiological evidence consistently links obesity with an increased risk of major cardiovascular outcomes, including coronary artery disease, myocardial infarction, and heart failure, as well as greater cardiovascular mortality compared with individuals of normal weight (Welsh et al., 2024; Khanna et al., 2025). In particular, the link between obesity and hypertension has grown harder to overlook. The percentage of adults with both obesity and hypertension in the United States increased from 39.6% in 2001 to 55.4% in 2023 (Tu et al., 2025), indicating a close epidemiological and mechanistic connection between high BP and excess body fat. This increasing trend highlights the importance of delineating the mechanisms that connect obesity to BP dysregulation for both prevention and development of more effective treatments (Tu et al., 2025). It is now evident that peripheral metabolic and vascular alterations alone cannot account for obesity-induced hypertension; central nervous system pathways that control BP also seem to be disrupted (Figure 1) (Hall et al., 2015; Seravalle and Grassi, 2017). Increased sympathetic nervous system (SNS) activity, renin-angiotensin-aldosterone system activation, impaired insulin signaling, and low-grade chronic inflammation are some of the interrelated mechanisms that have been linked to increased vascular resistance and sustained elevated BP (Thaler et al., 2012; Hall et al., 2015; Seravalle and Grassi, 2017). According to more recent research, neuroinflammatory and neurohormonal signaling have been implicated in the dysregulation of hypothalamic and brainstem circuits in obesity (Thaler et al., 2012; Guillemot-Legris and Muccioli, 2017; Patel et al., 2023). Determining the neurobiological factors that contribute to the relationship between obesity and hypertension is crucial for creating more effective interventions, given how common obesity has become and how closely it tracks with hypertension (Do Carmo et al., 2016).

One cell type that has drawn increasing attention in this context is the astrocyte. Available evidence supports a model in which obesity-associated metabolic and inflammatory signals may alter astrocyte homeostatic functions, there by altering neuronal excitability and autonomic regulation (Stern et al., 2016; O’Connor and Clark, 2018; Obara-Michlewska, 2022; Hasebe et al., 2024). The current review aims to bring together existing evidence on the neurogenic mechanisms behind obesity-induced hypertension, with special attention to how astrocytes contribute to central BP regulation, neuroinflammation and sympathetic activation.

## 2 Types of hypertension

One of the most modifiable risk factors for CVD is hypertension, which is defined as persistently elevated arterial blood pressure. The current ACC/AHA guidelines classify adult blood pressure (BP) to four groups: normal (systolic BP < 120 mmHg and diastolic <80 mmHg), elevated (systolic 120–129 mmHg and diastolic <80 mmHg), stage 1 hypertension (systolic 130–139 mmHg or diastolic 80–89 mmHg) and stage 2 hypertension (systolic ≥ 140 mmHg or diastolic ≥ 90 mmHg) (Jones et al., 2025). Clinicians can use this four-tier classification as a standard framework to help prevent, diagnose, and treat hypertension (Whelton et al., 2018).

### 2.1 Primary (essential) hypertension

With an estimated 90–95% of cases historically, primary or essential hypertension is by far the most prevalent type of the illness (Oparil et al., 2018). It develops because of a combination of inherited susceptibility alongside environmental and lifestyle factors that progressively impair the body’s capacity to maintain normal BP, rather than having a simple known cause. This process seems to involve a number of physiological systems, such as vascular function, renin-angiotensin-aldosterone signaling, SNS activity, and renal sodium handling (Oparil et al., 2018; Saxena et al., 2018). An individual’s risk of developing primary hypertension is shaped by a combination of modifiable factors. BP can rise over time due to a few factors, including being overweight, having metabolic problems like insulin resistance, consuming excessive amounts of sodium or alcohol, and physical inactivity. Age and individual genetic makeup further increase the risk (Carretero and Oparil, 2000; Valenzuela et al., 2021). Additionally, long term psychosocial stress may be involved, especially if it results in persistent activation of sympathetic and neuroendocrine pathways. Diet is important as well; low potassium intake has been associated with worse BP control, but there is less conclusive evidence regarding the role of dietary calcium (Carretero and Oparil, 2000; Houston and Harper, 2008; Kokubo et al., 2015).

### 2.2 Secondary hypertension

Secondary hypertension is estimated to occur in approximately 5–10% of adults with hypertension and arises when BP elevation can be attributed to a distinct underlying condition (Rimoldi et al., 2014). Recognizing a secondary cause of hypertension has important clinical implications, particularly in patients with features that warrant further investigation. Identifying the underlying cause of secondary hypertension can substantially influence clinical management, as cause-specific treatment may improve BP control and, in some patients, reduce or eliminate the need for chronic antihypertensive medication (Ott et al., 2013; Sarathy et al., 2022). Further evaluation for a secondary cause is particularly warranted when hypertension is resistant to standard therapy, appears early in life without typical predisposing factors, or shows an abrupt deterioration after previously stable BP control (Sarathy et al., 2022; Sudano et al., 2023). Additional red flags include severe hypertension and evidence of end-organ damage such as acute kidney injury, neurological complications, hypertensive retinopathy, flash pulmonary edema, or left ventricular hypertrophy as well as endocrine disorders like primary aldosteronism or Cushing’s syndrome (Rimoldi et al., 2014; Carey et al., 2018).

### 2.3 Neurogenic hypertension

When the brain’s central autonomic networks become unbalanced it leads to excessive sympathetic activity and persistent high arterial BP. This condition is known as neurogenic hypertension. The paraventricular nucleus (PVN), nucleus tractus solitarius (NTS), and rostral ventrolateral medulla (RVLM), among other important cardiovascular control regions in the hypothalamus and brainstem, collaborate to integrate neural and hormonal signals that ultimately influence sympathetic activity and BP (Lamptey et al., 2023). Instead of peripheral factors acting alone, this type of hypertension is caused by altered central neural control of autonomic function, which both initiates and maintains the rise in BP. An increasing amount of research suggest that neurogenic mechanisms play a significant role in hypertension in general, with increased SNS activity being a prominent characteristic of neurogenic hypertension. The central activation of angiotensin II signaling pathway appears to be particularly significant within key autonomic nuclei, serving as a crucial driver of sympathoexcitation that results in long-term increases in BP and vascular tone (Iyer et al., 1996; Feng et al., 2010; Sharma et al., 2021; Lamptey et al., 2023).

### 2.4 Obesity-induced hypertension (OIH)

Alarmingly, the global prevalence of obesity has nearly tripled since 1975; in 2016, more than 1.9 billion adults (39%) were classified as overweight, including over 650 million (13%) with obesity, according to the World Health Organization (Hall et al., 2021). The burden is even greater in the United States, where obesity prevalence increased from 30% in 1999–2000 to 42% in 2017–2018 (Hales et al., 2020). OIH, a highly prevalent phenotype of elevated BP, arises from excess adiposity and its associated metabolic, neurohumoral, and inflammatory disturbances, including sustained SNS activation, dysregulation of the renin-angiotensin-aldosterone system, insulin resistance, and chronic low-grade inflammation (Garrison et al., 1987; Hotamisligil, 2006; Hall et al., 2015). Although early studies of obesity-associated neuroinflammation primarily focused on the hypothalamus, emerging evidence demonstrates that inflammatory changes also involve other brain regions, including the hippocampus, cortex, brainstem, and amygdala (Lamptey et al., 2023). The association between obesity and hypertension has been well described (Hall et al., 2015). Large population-based studies demonstrate an almost linear relationship between body mass index (BMI) and BP, indicating that increasing adiposity is accompanied by progressive elevations in arterial pressure (Wang and Wang, 2004; Gelber et al., 2007). Findings from the Framingham heart study further highlight this link, showing that approximately 78% of essential hypertension cases in men and 65% in women are attributable to obesity (Garrison et al., 1987). Epidemiological studies indicate that 60–70% of adults with obesity develop hypertension, and individuals with obesity are two to three times more likely to develop hypertension compared with normal-weight individuals (Wang and Wang, 2004; Gelber et al., 2007). Given the continued global rise in obesity prevalence, OIH is expected to remain a major contributor to the growing burden of CVD worldwide (Gelber et al., 2007; Dorresteijn et al., 2011; World Health Organization, 2017; Phelps et al., 2024).

### 2.5 Stress-induced hypertension

Through recurrent, brief increases in BP and persistent activation of neuroendocrine pathways that encourage vasoconstriction, psychological stress plays a role in the development of hypertension (Kulkarni et al., 1998). An increasing amount of research demonstrates how psychosocial stressors, such as personality traits, mental health issues, housing instability, social isolation, occupational stress, and poor sleep quality, influence an individual’s BP responses (Cuffee et al., 2014). Stress responses vary markedly among individuals and are influenced by personality, prior experience, and coping capacity, but are uniformly characterized by activation of the SNS and the hypothalamic–pituitary–adrenal axis (Selye, 1950; Munakata, 2018). These systems support short-term adaptation through allostasis; however, excessive or prolonged activation becomes maladaptive (McEWEN, 1998). Heightened BP reactivity to mental stress has been associated with increased vascular damage, particularly in the presence of arterial stiffness and impaired natriuretic responses (Lipman et al., 2002; Gerber and Pühse, 2009; Harshfield et al., 2009). Persistent elevation of BP, endothelial dysfunction and increased shear stress are caused by prolonged activation of the SNS, renin-angiotensin-aldosterone system, and hypothalamic–pituitary–adrenal axis under chronic stress conditions. Endothelial homeostasis and vascular structure are further compromised by stress-induced neuroendocrine activation, which also encourages inflammatory signaling (Black and Garbutt, 2002; Puzserova and Bernatova, 2016; Ushakov et al., 2017). Chronic psychological stress also raises the risk of harmful lifestyle choices like smoking, excessive alcohol consumption (McQueen and Celentano, 1982), physical inactivity, depression (Artinian et al., 2006), and insomnia (The VA Mid-Atlantic Mental Illness Research Education and Clinical Center Registry Workgroup et al., 2015) all of which increase the risk of hypertension.

### 2.6 Salt induced hypertension

A significant rise in BP in reaction to dietary sodium intake is known as salt-sensitive hypertension. Central sodium sensing, reduced renal sodium excretion, sympathetic activation, and functional changes in brain areas like the organum vasculum of the lamina terminalis and the subfornical organ are all parts of this phenotype (Weinberger, 1996; Feng Earley et al., 2025). The exact mechanisms underlying salt-induced hypertension are still unclear, even though the relationship between dietary sodium and BP has been thoroughly investigated. Increased salt consumption causes BP to rise, especially in salt-sensitive people whose normal adaptive natriuretic and neurohormonal responses to sodium loading are compromised. Overconsumption of sodium alters the renin-angiotensin-aldosterone system’s regulation, interferes with pressure–natriuresis mechanisms, and encourages fluid retention, all of which raise cardiac output and vascular resistance (Guyton et al., 1972; Guyton, 1991; Weinberger, 1996; Drenjančević-Perić et al., 2011). Beyond these effects on the kidneys, new research suggests that a high salt diet may also affect vascular reactivity and SNS activity, which would further contribute to the maintenance of hypertension (Feng Earley et al., 2025). Together, these interconnected alterations result in a maladaptive state where vascular tone and sodium balance become dysregulated leading to a persistent rise in arterial pressure (Drenjančević-Perić et al., 2011).

### 2.7 Hypertension in pregnancy (pre-eclampsia)

Hypertension in pregnancy encompasses several clinical conditions, most commonly pregnancy-induced hypertension and pre-eclampsia. Pregnancy-induced hypertension is characterized by a rise in BP during the second half of pregnancy without proteinuria, whereas pre-eclampsia is a multisystem disorder unique to pregnancy that typically occurs after 20 weeks of gestation and is marked by hypertension accompanied by proteinuria and systemic complications (Brown et al., 2018). Pregnancy-induced hypertension affects approximately 10% of pregnancies, while pre-eclampsia complicates 2–8% of pregnancies worldwide (World Health Organization, 2011; Brown et al., 2018). Although the precise etiology of pre-eclampsia remains unclear, it is widely considered a multifactorial condition associated with abnormal placental implantation and impaired placental perfusion during early pregnancy (Roberts and Hubel, 2009). The risk of developing pre-eclampsia is increased among women with conditions linked to microvascular dysfunction, including diabetes, chronic hypertension, and collagen vascular diseases, as well as in those with multiple pregnancies (ACOG, 2020). Additional contributing factors such as genetic susceptibility, increased maternal age, and higher parity have also been implicated in the development of hypertensive disorders during pregnancy (Brown et al., 2018).

### 2.8 Hypertension in obstructive sleep Apnea (OSA)

Recurrent episodes of upper airway obstruction during sleep are the hallmark of OSA, a sleep related breathing disorder. These episodes which can result in either a complete cessation of airflow (apnea) or a partial reduction (hypopnea), are typically characterized by a minimum frequency of five episodes per hour, each lasting at least 10 s. Due to decreased or nonexistent ventilation, these episodes cause intermittent hypoxia and hypercapnia. OSA is the most common type of sleep apnea, though there are other types as well, such as central and mixed types (Mirrakhimov, 2012; Patel et al., 2019). Compared to the general public, OSA is more prevalent among individuals with hypertension, and many patients with OSA also exhibit elevated BP (Floras, 2015). OSA and hypertension are closely related, and research indicates that breathing disorders during sleep may play a role in the onset and maintenance of hypertension. Notably, 75% of patients with treatment resistant hypertension have OSA, highlighting its important role in resistant BP elevation (Mashaqi and Gozal, 2019). Increased sympathetic activity, peripheral vasoconstriction, activation of the renin-angiotensin-aldosterone system, and compromised baroreceptor reflexes are some of the mechanisms involved in the complex pathophysiology of hypertension in OSA (Ahmad et al., 2017). Patients with OSA may also develop hypertension due to autonomic imbalance, nocturnal fluid shifts, intermittent hypoxemia, and poor sleep quality (Bangash et al., 2020).

## 3 Mechanisms linking obesity to hypertension

Increasing adiposity is consistently associated with higher BP in a variety of populations, indicating a strong link between obesity and the development of hypertension. Neural, renal, metabolic, and vascular mechanisms interact to produce the multifactorial pathogenesis of OIH. The SNS, altered renal sodium handling, hormonal dysregulation, endothelial dysfunction, and chronic inflammation are some of the interrelated pathways that sustain elevated arterial pressure in individual with obesity (Kotchen, 2010; Kotsis et al., 2010).

Increased SNS activity is one of the key mechanisms connecting obesity to hypertension, as shown in Figure 2. Increased sympathetic tone, which causes vasoconstriction, increases heart rate and augments renal sodium reabsorption. Collectively, these factors contribute to the elevated BP frequently observed in individuals with obesity. This sympathetic overactivation can be further increased by a number of metabolic signals associated with excess adiposity, including increased circulatory free fatty acids, insulin and leptin (Kotsis et al., 2010).

**Figure 2:**
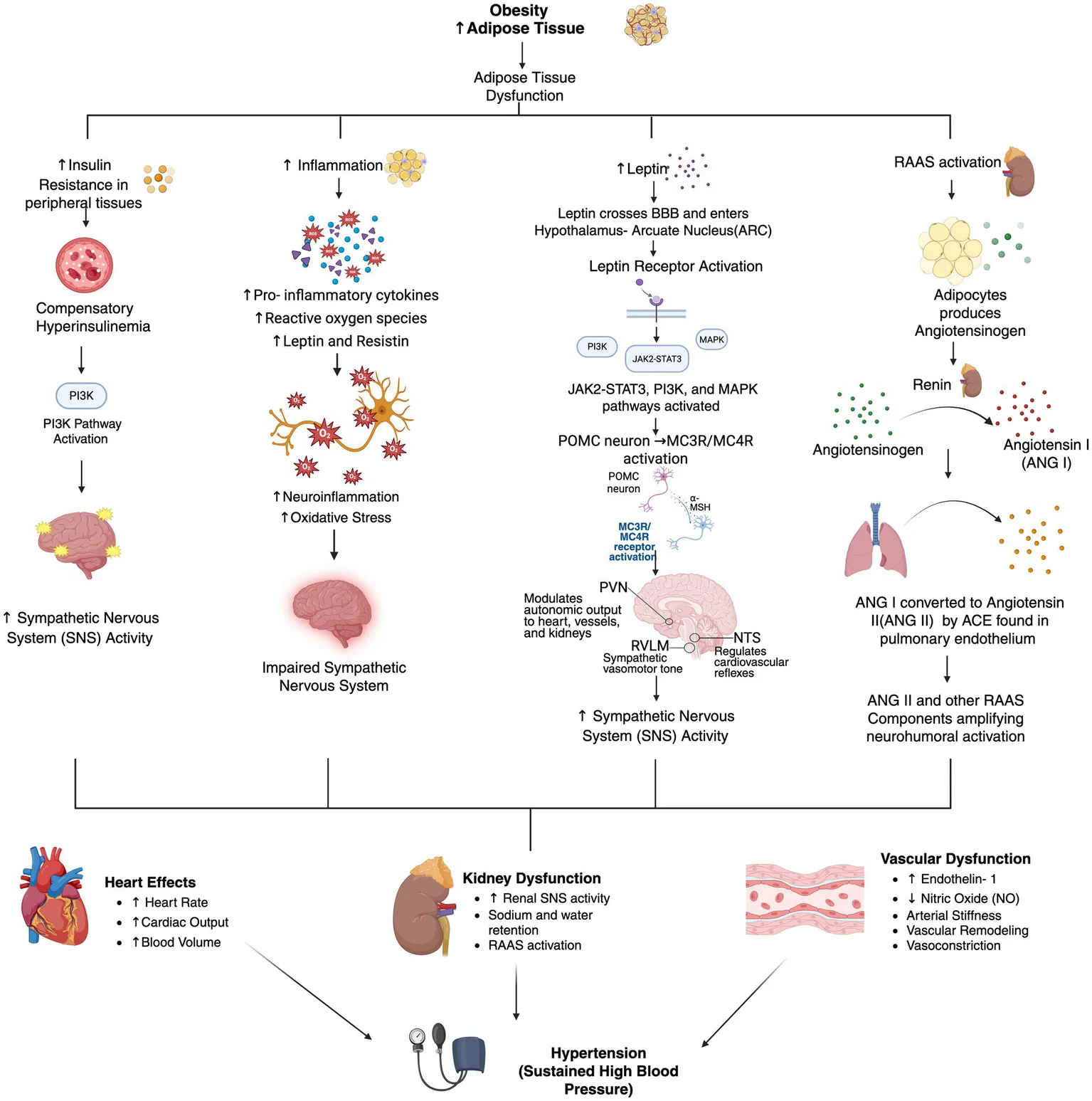
Mechanisms linking obesity to sympathetic activation and hypertension. Obesity-associated adipose tissue dysfunction promotes multiple interconnected pathways that contribute to increased sympathetic nervous system (SNS) activity and sustained elevation of blood pressure. Insulin resistance and compensatory hyperinsulinemia enhance sympathetic activation through PI3K-dependent signaling. Increased systemic inflammation, pro-inflammatory cytokines, reactive oxygen species, and leptin promote neuroinflammation and oxidative stress, resulting in dysregulation of central autonomic pathways. Elevated circulating leptin crosses the blood–brain barrier and activates leptin receptors in the hypothalamic arcuate nucleus (ARC), engaging JAK2-STAT3, PI3K, and MAPK signaling and downstream POMC-MC3R/MC4R pathways that influence autonomic nuclei, including the PVN, RVLM, and NTS, thereby increasing sympathetic outflow. In parallel, obesity activates the renin-angiotensin-aldosterone system (RAAS), further enhancing neurohumoral and sympathetic activation. Collectively, increased sympathetic drive, renal sodium and water retention, cardiac stimulation, and vascular dysfunction contribute to the development and maintenance of obesity-induced hypertension.

Additionally, renal mechanisms are central in obesity-associated hypertension (Figure 2). Increased renal tubular sodium reabsorption in obesity causes extracellular fluid volume expansion and sodium retention. Because of this change, the pressure-natriuresis relationship is reset toward higher BP levels, requiring a higher arterial pressure to maintain proper sodium balance.(Hall et al., 2010; Kotsis et al., 2010).

Another important contributor is activation of the renin-angiotensin-aldosterone system. Obesity is associated with increased circulating levels of renin, angiotensin II, and aldosterone, which enhance vasoconstriction, sodium retention, and vascular remodeling. In addition, adipose tissue itself can produce angiotensinogen and other components of the renin-angiotensin-aldosterone system, further amplifying neurohumoral activation and contributing to hypertension (Kotchen, 2010; Kotsis et al., 2010).

As shown in Figure 2, metabolic abnormalities, especially insulin resistance and hyperinsulinemia, also contribute to elevated BP in obesity. Impaired vasodilation, elevated sympathetic activity, and increased renal sodium retention are interconnected to insulin resistance. These effects raise peripheral resistance and encourage vascular dysfunction, two important aspects of the development of hypertension in obese individuals (Kotsis et al., 2010; DeMarco et al., 2014).

These processes are further exacerbated by adipokines and inflammatory mediators secreted by dysfunctional adipose tissue. Reactive oxygen species, pro-inflammatory cytokines, and adipokines like resistin and leptin are all elevated in obesity. These molecules are involved in endothelial dysfunction, oxidative stress, and systemic inflammation (Figure 2). The balance between vasodilation and vasoconstriction is disrupted by decreased nitric oxide availability and increased endothelin-1 production, which raises vascular resistance and encourages hypertension (Kotsis et al., 2010; Dorresteijn et al., 2012).

Lastly, elevated BP in obesity is also a result of structural and functional alterations in the vasculature. Endothelial dysfunction, arterial stiffness and vascular remodeling are caused by oxidative stress, neurohumoral activation, and chronic inflammation. These changes exacerbate hypertension by impairing regular vascular regulation and raising peripheral resistance. Overall, these mechanisms show that obesity-associated hypertension is caused by a complex interconnection of neural, renal, metabolic, and vascular factors than a single pathological pathway (Kotsis et al., 2010; Dorresteijn et al., 2012).

## 4 Central brain signaling pathways linking obesity to hypertension

It is more widely recognized that OIH is a condition caused by dysregulation of the central nervous system (CNS), because metabolic signals from adipose tissue interact with neural circuits that control sympathetic outflow and cardiovascular function (Figure 3). Elevated levels of inflammatory mediators, hormones, and adipokines in the blood are caused by excessive obesity. These substances all affect the parts of the brain involved in autonomic regulation of BP. Leptin is a hormone derived from adipocytes that increases in proportions to fat mass. Leptin is one of key signals that is crucial in linking obesity to neurogenic hypertension, as it activates central pathways that increase BP and SNS activity (Figure 3) (Esler et al., 2008; Hall et al., 2015; Bell and Rahmouni, 2016; Do Carmo et al., 2016).

**Figure 3:**
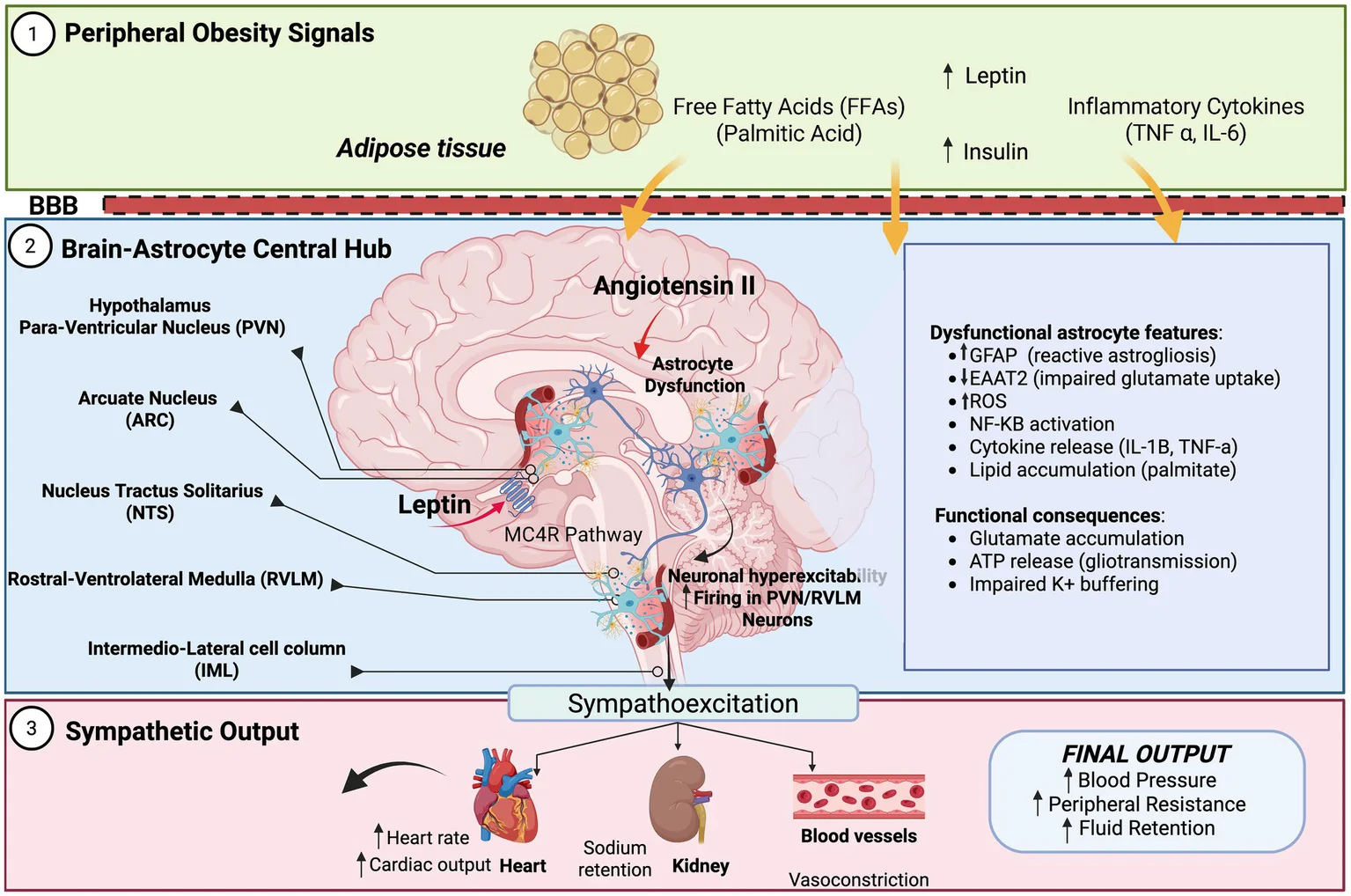
Astrocyte-mediated central mechanism in obesity-induced hypertension. Obesity-derived metabolic and inflammatory signals, including leptin, insulin, free fatty acids, and cytokines, cross the blood–brain barrier and activate hypothalamic and brainstem circuits. Astrocytes in these regions undergo reactive transformation characterized by increased GFAP expression, impaired glutamate uptake (EAAT2 dysfunction), oxidative stress, and inflammatory signaling. These changes promote neuronal hyperexcitability within autonomic regulatory nuclei such as the paraventricular nucleus and rostral ventrolateral medulla, leading to enhanced sympathetic outflow (sympathoexcitation) and sustained elevation of arterial pressure.

The hypothalamus, especially the arcuate nucleus (ARC), serves as an important site for connecting peripheral metabolic signals. Pro-opiomelanocortin and agouti-related peptide neurons, which are involved in the control of energy balance, sympathetic activity, and cardiovascular function, are found in substantial populations in the ARC (Rahmouni and Morgan, 2007). In obesity, elevated leptin levels activate ARC leptin receptors on these hypothalamic neurons, triggering downstream signaling cascades, which modulate neuronal excitability and sympathetic activity. Activation of pro-opiomelanocortin neurons stimulates the release of α-melanocyte-stimulating hormone, which acts on melanocortin-3 and melanocortin-4 receptors (MC3R/MC4R) in downstream autonomic nuclei to increase sympathetic nerve activity and BP (Figure 3) (Kalil and Haynes, 2012; Do Carmo et al., 2013).

As shown in Figure 3, these hypothalamic signals are transmitted to brainstem cardiovascular regulatory centers, including the PVN, NTS, and RVLM. By combining metabolic and neuroendocrine inputs and projecting to sympathetic premotor neurons, the PVN regulates autonomic output to the heart, vasculature, and kidneys. The RVLM is a significant regulator of sympathetic vasomotor tone (Sivasubramanian et al., 2026). The NTS integrates baroreceptor and chemoreceptor signals to regulate cardiovascular reflexes (Do Carmo et al., 2016). Leptin signaling within these nuclei has been shown in experiments to raise arterial pressure and renal sympathetic nerve activity, underscoring their role in obesity-related neurogenic hypertension (Laule and Rahmouni, 2025).

A unique feature of obesity is selective leptin resistance, in which leptin’s metabolic actions on appetite and energy expenditure become impaired, while its ability to stimulate sympathetic outflow remains preserved. This selective preservation of cardiovascular sympathetic effects contributes to chronic sympathetic overactivation in obesity. Elevated sympathetic activity increases renal sodium reabsorption, enhances vascular resistance, and promotes activation of the renin-angiotensin-aldosterone system, collectively leading to sustained hypertension (Da Silva et al., 2013; Laule and Rahmouni, 2025).

Obesity is connected to oxidative stress, glial activation and hypothalamic inflammation in addition to leptin signaling. All those processes reduce neuronal control of cardiovascular function. These neuroinflammatory changes can increase sympathetic drive and contribute to long term BP elevations. Overall, these findings highlight that brain signaling pathways particularly those involving hypothalamic-brainstem autonomic networks and leptin-melanocortin circuits, play an important role in the development of OIH (Do Carmo et al., 2016).

## 5 Role of astrocytes in obesity-induced hypertension

Astrocytes, the most abundant glial cell population in the CNS, are widely recognized as critical regulators of neurovascular (Abbott et al., 2010; Huang et al., 2019) and neuroendocrine function; roles that go beyond their traditional supportive functions (Gradisnik and Velnar, 2023). In the context of obesity, astrocytes emerge as key mediators linking metabolic disturbances to central autonomic dysregulation, as illustrated in Figure 4, Stern et al. (2016), Obara-Michlewska (2022). Excess nutrient availability, particularly elevated circulating saturated fatty acids such as palmitic acid, along with adipokines and inflammatory mediators, can access the brain and induce astrocyte reactivity (often termed astrogliosis) within key autonomic regions, including the hypothalamus and brainstem (De Souza et al., 2005; Milanski et al., 2009; Horvath et al., 2010). This reactive astrocytic phenotype is characterized by morphological remodeling, increased expression of glial fibrillary acidic protein (GFAP), and the release of pro-inflammatory cytokines, collectively contributing to a state of chronic neuroinflammation (Milanski et al., 2009; Obara-Michlewska, 2022; Firth et al., 2024).

**Figure 4:**
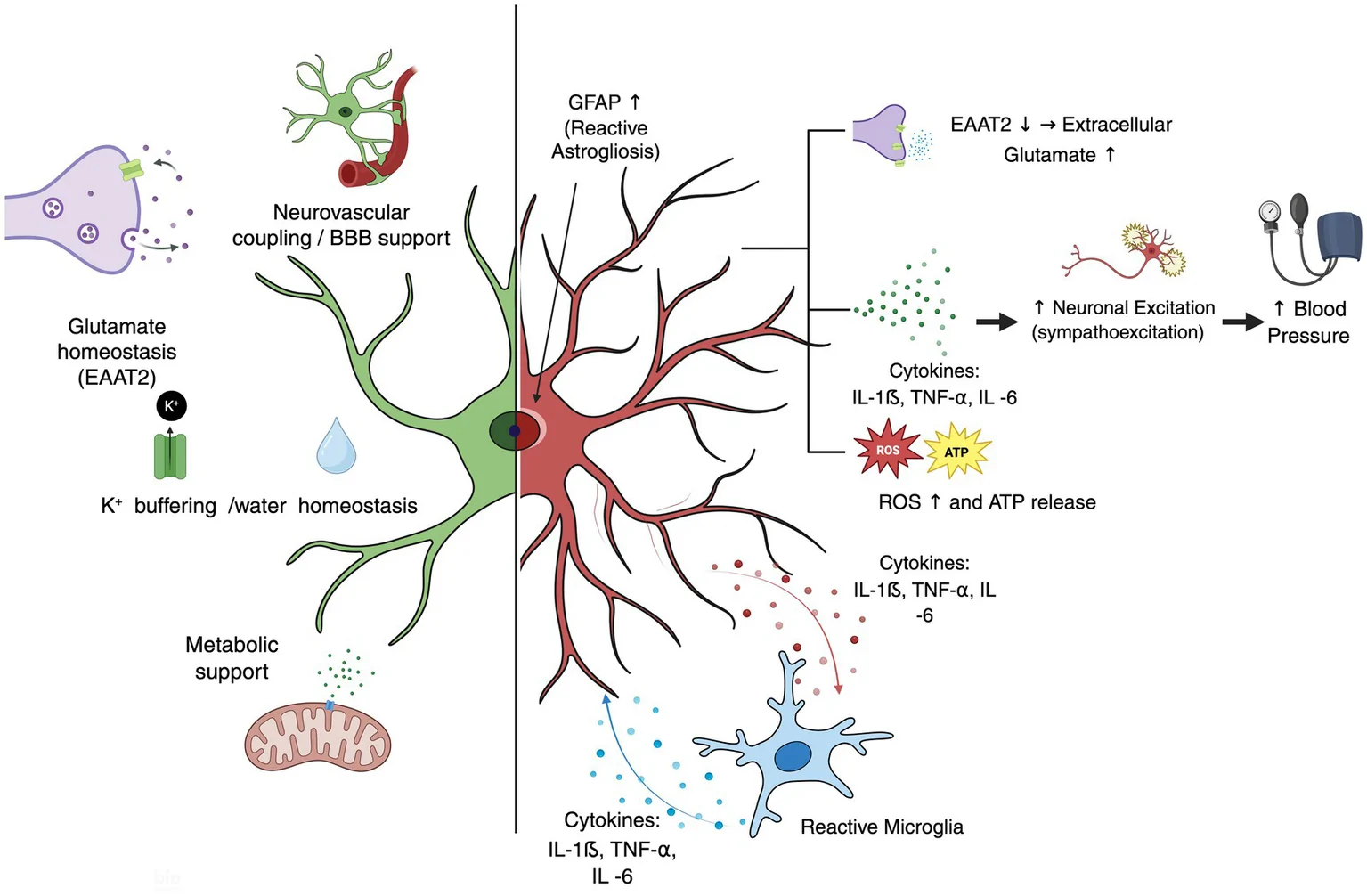
Role of astrocytes in the central nervous system compared to their role in obesity-induced hypertension. Under physiological conditions, astrocytes maintain glutamate homeostasis, potassium buffering, metabolic support, and neurovascular regulation. Obesity induces reactive astrogliosis characterized by increased GFAP expression, reduced EAAT2-mediated glutamate uptake, cytokine release, and oxidative stress. Interactions between reactive astrocytes and microglia amplify neuroinflammation, increase neuronal excitability and sympathetic outflow, and contribute to the development of obesity-induced hypertension.

As shown in Figure 4, astrocytes play a pivotal role in maintaining synaptic homeostasis through the regulation of extracellular glutamate, potassium buffering, and metabolic support to neurons (Goenaga et al., 2023; Kruyer et al., 2023). However, under obesogenic conditions, these homeostatic functions become disrupted (Joseph et al., 2025). Glutamate is the principal excitatory neurotransmitter in the CNS, mediating most of the fast synaptic transmission. To activate postsynaptic glutamate receptors, it diffuses across the synaptic cleft after being released from presynaptic terminals (Bjørnsen et al., 2014). Excitatory amino acid transporters (EAATs) tightly control the cessation of glutamatergic signaling by rapidly eliminating extracellular glutamate and avoiding excitotoxicity. To date, five mammalian EAAT isoforms (EAAT1–5) have been identified (Yamada et al., 1996; Arriza et al., 1997; Dehnes et al., 1998; Holmseth et al., 2012; Bjørnsen et al., 2014), with EAAT1 and EAAT2 predominantly expressed in astrocytes and localized to the plasma membrane, whereas EAAT3, EAAT4, and EAAT5 are primarily neuronal (Takahashi et al., 2015). Glutamate transported into astrocyte is efficiently converted into the non-neuroactive amino acid glutamine by the astrocyte-specific enzyme glutamine synthetase (GS). After being exported from astrocytes, extracellular glutamine is imported into neuronal terminals via sodium-coupled neutral amino acid transporters (SNATs) and subsequently converted back into glutamate by phosphate-activated glutaminase (PAG). This glutamate recycling pathway is called glutamate-glutamine cycle. Impaired astrocytic glutamate uptake primarily mediated by EAAT2 leads to elevated extracellular glutamate levels, promoting neuronal hyperexcitability and excitotoxic signaling (Rothstein et al., 1996; Bjørnsen et al., 2014; Takahashi et al., 2015).

In parallel, astrocyte-derived gliotransmitters, including ATP and D-serine, further modulate synaptic transmission and might increase excitatory drive within autonomic circuits (Harada et al., 2015). This dysregulation is important in cardiovascular regulatory regions such as PVN and RVLM where increased excitatory signaling can enhance sympathetic outflow (Horvath et al., 2010; Stern et al., 2016; Balasubramanian et al., 2020; Patel et al., 2023; Verma and Feng Earley, 2025).

In addition to neurotransmitter imbalance, astrocytes are also involved in metabolic and inflammatory signaling pathways that change neuronal activity. Exposure to saturated fatty acids, such as palmitic acid, causes endoplasmic reticulum stress, mitochondrial dysfunction, increased production of reactive oxygen species (Saipuljumri et al., 2026) and lipid accumulation in astrocytes under obesogenic conditions (Tse et al., 2018; Sánchez-Alegría and Arias, 2023; Zhao et al., 2024). These cellular alterations can trigger the activation of inflammatory pathways such as NF-κB signaling, which in turn can increase the release of cytokines like TNF-α and IL-1β (Balasubramanian et al., 2020; Zhao et al., 2024). In addition to interfering with neuronal signaling, this astrocyte-driven neuroinflammation increases the activity of the central renin-angiotensin-aldosterone system and reduces baroreflex sensitivity, all of which contribute to prolonged sympathoexcitation and high BP (O’Connor and Clark, 2018).

Astrocytes play a crucial role in neurovascular coupling and blood brain barrier (BBB) integrity (Figure 4) (Huang et al., 2019; Díaz-Castro et al., 2023), both of which become disrupted in the context of obesity. In addition to increasing BBB permeability and impairing cerebral blood flow regulation, dysfunctional astrocytic endfeet can facilitate the entry of circulating inflammatory mediators and metabolic substrates into the brain (Huang et al., 2025). This feed-forward loop exacerbates central inflammation and autonomic dysregulation. Additionally, astrocytes influence neuronal circuits through energy metabolism and lactate shuttling, processes that may differ in obesity and lead to abnormal neuronal activation (Bonvento and Bolaños, 2021; Roumes et al., 2023) (see Figure 5).

**Figure 5:**
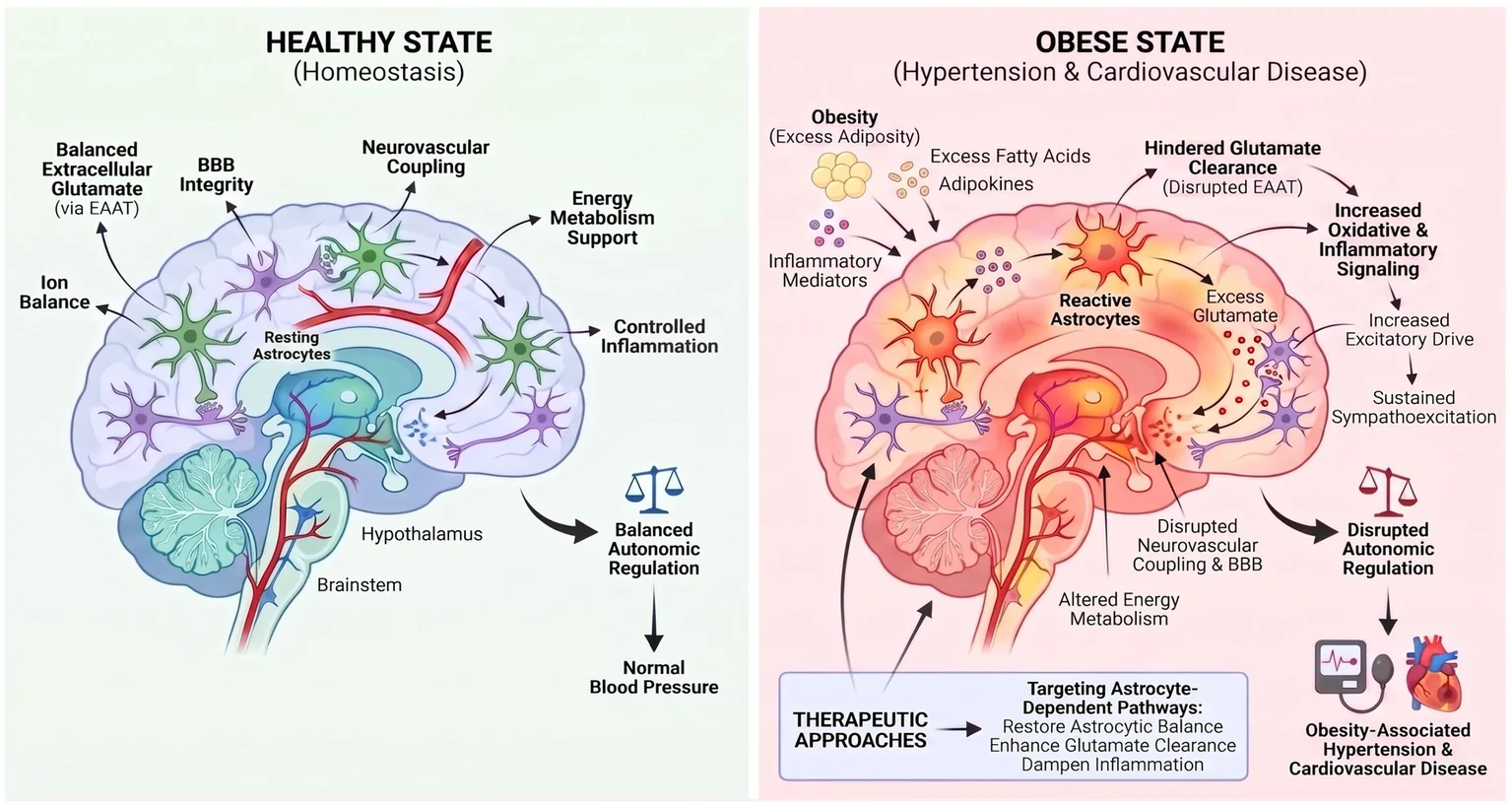
Proposed role of astrocyte dysfunction in obesity-associated hypertension and cardiovascular disease.Under healthy conditions (left), astrocytes maintain extracellular glutamate and ion homeostasis, blood–brain barrier integrity, neurovascular coupling, and neuronal metabolic support, contributing to balanced autonomic regulation and normal blood pressure. In obesity (right), excess fatty acids, adipokines, and inflammatory mediators promote reactive astrogliosis, impaired glutamate clearance, and disrupted neurovascular and metabolic functions. These alterations may enhance excitatory signaling within autonomic circuits, including the hypothalamus and brainstem, contributing to sustained sympathoexcitation, hypertension, and cardiovascular disease. Potential therapeutic approaches include restoring astrocytic glutamate transport, reducing inflammation, and improving astrocyte function. BBB, blood–brain barrier; EAAT2, excitatory amino acid transporter 2.

Overall, these findings point to astrocytes as key integrators of neural, inflammatory, and metabolic signals in OIH. However, direct causal evidence that astrocyte dysfunction initiates or sustains sympathetic activation and hypertension specifically in OIH remains limited. Astrocytes contribute to the ongoing activation of sympathetic pathways that result in elevated arterial pressure by regulating synaptic activity, neuroinflammation, and neurovascular function (Stern et al., 2016; Obara-Michlewska, 2022; Firth et al., 2024; Verma and Feng Earley, 2025). Targeting astrocyte-specific mechanisms such as restoration of glutamate transport, attenuation of inflammatory signaling, and modulation of lipid-induced astrocytic dysfunction may therefore represent a promising therapeutic strategy for mitigating neurogenic pathways of obesity-associated hypertension (Huang et al., 2025; Yang et al., 2025).

## 6 Astrocyte-targeted therapeutic strategies for obesity-induced hypertension

Increased attention has focused on different therapeutic approaches to manage and mitigate obesity-related hypertension (Huang et al., 2025; Yang et al., 2025). While there are many mechanisms involved in the progression of hypertension in obesity, a promising area of research includes astrocyte-specific mechanisms, such as restoring glutamate homeostasis and attenuating inflammatory signaling. These mechanisms may be targeted by experimental agents and, potentially by clinically approved weight-lowering therapies such as GLP-1 based drugs.

Neuroinflammation is a main characteristic of OIH that has the highest potential for therapeutic intervention. High-fat diets and obesity activate inflammatory pathways through toll-like receptors, microglia-induced activation, cytokine production, oxidative stress, and the overactivation of the SNS (Milanski et al., 2009; Sonnefeld et al., 2023). The IKKβ/NF-κB plays an important role in the neuroinflammation process, driving the production of pro-inflammatory cytokines TNF-α, IL-1β, and IL-6 (Zhang et al., 2017; Bhusal et al., 2022). In addition to being a pro-inflammation pathway, it also promotes reactive astrogliosis, activates microglia consequently leading to amplifying neuroinflammation, and increasing oxidative stress (Sorg et al., 1997; Lee and Jeong, 2016; Zhang et al., 2017; Karki et al., 2018; Bhusal et al., 2022; Huang et al., 2025). Within its effects on triggering reactive astrogliosis, alterations on astrocyte plasticity can negatively affect the astrocyte’s interactions with neurons and blood vessels causing detrimental effects on BP, glucose homeostasis, and body weight leading to obesity (Zhang et al., 2017).

One of the newly studied therapeutic treatments that targets multiple astrocyte-specific mechanisms causing OIH is arundic acid (Karki et al., 2018). Under normal conditions, astrocytes maintain excitatory neurotransmitter homeostasis through glutamate transporters, EAAT1 and EAAT2, by removing the excess glutamate in the extracellular space (Bjørnsen et al., 2014). When there is an impairment of this mechanism, accumulation of glutamate in the extracellular space causes neuronal excitability, contributing to a SNS overactivity. In this regard, studies have shown that arundic acid increases glutamate transporter EAAT1 expression and glutamate uptake by activating ERK, Akt, and NF-κB signaling pathways (Ishibashi et al., 2007; Karki et al., 2018; Cordeiro et al., 2020; Sonnefeld et al., 2023). In addition to the glutamate clearance, it also suppresses the excess production of astrocytic production of pro inflammatory mediators such as S100B, reducing oxidative stress, neuroinflammation, attenuating reactive astrogliosis (Sorg et al., 1997; Moghaddam et al., 2014; Cordeiro et al., 2020; Barros et al., 2022; Sonnefeld et al., 2023). Even though arundic acid is yet to be available for commercial use, various clinical trials and pharmacological studies are being performed with mice to fully understand the efficacy as a potential neuroprotective drug (Ishibashi et al., 2007).

Beyond astrocyte-specific modulation, targeting broad inflammatory pathways may provide additional therapeutic benefits to treat neuroinflammation. Butein, a dietary flavonoid derived from the bark of *Rhus vermicelli,* acts as a potent anti-inflammatory and neuroprotective agent by inhibiting the IKK/NF-κB signaling pathway (Lee and Jeong, 2016). By inhibiting this pathway, butein effectively suppresses the production of pro-inflammatory cytokines such as TNF- α, IL-1β, and IL-6 (Karki et al., 2018; Bhusal et al., 2022; Sonnefeld et al., 2023). Butein also activates the Nrf2/ARE pathways that trigger the expression of heme oxygenase-1 (HO1), an antioxidant enzyme that protects cells from oxidative stress and reactive oxygen species (Lee and Jeong, 2016). Similar reductions in oxidative stress and astrogliosis have been observed with berberine, an alkaloid found in plants from the berberidaceae family, suggesting that therapies targeting both inflammation and oxidative damage may provide neuroprotective benefits (Moghaddam et al., 2014).

In contrast to these experimental compounds, GLP-1 receptor agonists are already in clinical use and may intersect with the same astrocytic pathways (Timper et al., 2020; Du et al., 2026; Russjan et al., 2026). Semaglutide, liraglutide, and the dual GIP/GLP-1 receptor agonist tirzepatide reduce body weight and BP in people with obesity. GLP-1 receptors are expressed in hypothalamic and brainstem region involved in autonomic control, including the NTS, and GLP-1 receptor signaling has been reported to attenuate reactive gliosis and neuroinflammation, with GLP-1 receptors described on astrocytes (Timper et al., 2020). However, central GLP-1 receptor activation has also been associated with increased sympathetic activity and heart rate in some experimental and clinical settings, so the final autonomic effects of these drugs are not fully determined. It also remains unknown whether their BP-lowering effects arise solely from weight loss or involve weight-independent astrocytic mechanisms, such as restoration of EAAT2-mediated glutamate clearance or suppression of IKK/NF-κB dependent inflammation, as described above (Du et al., 2026; Russjan et al., 2026).

While the development of novel therapies to treat OIH offers promising treatment options, lifestyle interventions remain the cornerstone of disease prevention (Narkiewicz, 2006; Antza et al., 2017). Reducing the consumption of high-fat diets can attenuate the previously mentioned mechanisms of neuroinflammation, oxidative stress, and metabolic dysfunction that contribute to SNS overexcitation (Narkiewicz, 2006; Antza et al., 2017; Bhusal et al., 2022; Sonnefeld et al., 2023). For individuals presenting with clinical disease, dietary modifications such as consuming antioxidant-rich food or antioxidant-based interventions may help mitigate the effects of oxidative stress and neuroinflammation, thereby preserving astrocyte function and controlling disease progression (Sorg et al., 1997; Moghaddam et al., 2014; Lee and Jeong, 2016). Ultimately, prevention through a balanced diet remains the most effective and accessible approach for reducing the risk of obesity and its associated complications.

## 7 Current knowledge gaps and future directions

The precise role of astrocytes in OIH is still largely unknown. Although astrocytes regulate neurotransmitter homeostasis, inflammatory signaling, gliotransmission, and metabolic support within cardiovascular regulatory regions such as the PVN, NTS, and RVLM, most available evidence comes from studies of hypothalamic metabolic regulation or other models of hypertension. Thus, the mechanisms by which obesity-associated astrocytic dysfunction contributes specifically to sympathoexcitation and elevated BP remain unclear.

A substantial knowledge gap remains regarding the regional and molecular heterogeneity of astrocytes within metabolic and cardiovascular regulatory centers. Recent single-nucleus RNA sequencing identified distinct RVLM astrocyte populations and demonstrated that increased contactin-associated protein-like 2 (Cntnap2) expression reduces EAAT2 expression and glutamate uptake, leading to increased neuronal excitability, sympathetic outflow, and BP in spontaneously hypertensive rats (Zhang et al., 2026). These findings provide evidence that specific astrocytic populations can directly influence cardiovascular function. However, whether obesity produces similar astrocyte-specific molecular changes in the RVLM or other autonomic nuclei remains unknown. Studies employing single-cell or single-nucleus RNA sequencing, proteomics, lipidomics, metabolomics, and spatial transcriptomics may help to identify astrocyte subpopulations that change during OIH and determine whether these populations have adaptive or pathological effects.

A crucial unanswered question is whether obesity-associated metabolic stress impairs astrocytic glutamate clearance and thereby drives sympathoexcitation. Given that reduced astrocytic EAAT2 expression in the RVLM and its association with neuronal excitability and hypertension (Zhang et al., 2026), it remains unclear whether obesity-associated metabolic factors directly impair EAAT2. Elevated palmitic acid alters astrocytic metabolism and disrupts pathways involving the glutamate-glutamine cycle (Sivasubramanian et al., 2024), supporting the need to determine whether metabolic stress-induced EAAT2 dysfunction precedes glutamate accumulation, sympathoexcitation, and hypertension.

The mechanisms by which inflammation linked to obesity modifies astrocytic function represent another critical gap. Consuming a high-fat diet causes reactive gliosis and hypothalamic inflammation. The development and maintenance of diet-induced hypothalamic inflammation and metabolic dysfunction have been linked to astrocytic IKKβ/NF-κB signaling (Douglass et al., 2017). In contrast, relatively little is known about inflammatory signaling in the brainstem cardiovascular centers during obesity. Future research should therefore determine whether NF-κB and associated inflammatory pathways are activated by metabolic stress in RVLM astrocytes, and whether this contributes to EAAT2 dysfunction and altered glutamate homeostasis.

The temporal relationship between astrocyte dysfunction and the onset of hypertension is also unclear. Because diet-induced inflammation and gliosis can appear early during high-fat feeding, potentially before some systemic metabolic effects of obesity (Thaler et al., 2012; Sivasubramanian et al., 2024), astrocytic changes could either precede or follow hypertension. Longitudinal studies combining radiotelemetric BP monitoring with measurements of sympathetic activity, and region-specific molecular characterization of astrocytes at different stages of high-fat feeding would answer this question. Such designs could also be paired with weight-lowering interventions, including GLP-1 receptor agonists, to distinguish astrocytic changes that depend on weight loss from those that do not (Timper et al., 2020).

Astrocyte communication with other neural and non-neural cell populations is another crucial area that needs research. Obesity-associated neuroinflammation does not arise from astrocyte alone; astrocytes, microglia, neurons, endothelial cells, and peripheral immune signals interact intricately (Kälin et al., 2015; Rahman et al., 2018). Cell-specific genetic modification combined with simultaneous assessment of astrocytes, microglia, neurons, and vascular cells will be required to determine whether astrocytes initiate pathological signaling or amplify inflammatory signals generated by surrounding cells. In this it will also be interesting to examine the role of extracellular vesicles in mediating these communications between the cells.

Another significant limitation of the current literature is the lack of investigation into sex differences. Male animals have been used in many experimental studies examining obesity-related neuroinflammation and neurogenic hypertension, which limits the generalizability of their results. Recent studies have shown that astrocytes react to saturated fatty acids in a sex-dependent way. In hypothalamic astrocytes from male and female mice, palmitic acid causes different metabolic reactions such as variations in the glutamate-glutamine cycle and energy metabolism pathways (Collado-Perez et al., 2024). Similar to this, human astrocytes exhibit sex-specific susceptibility to lipotoxicity caused by palmitic acid, with variations in oxidative stress, antioxidant responses, and steroid hormone related pathways (Hidalgo-Lanussa et al., 2024). Whether astrocytic responses to GLP-1 receptor activation also differ by sex is unknown. These findings emphasize the importance of including both sexes in future studies.

Translation to human OIH remains another major challenge because most mechanistic evidence is derived from rodents or cultured cells. Human neuroimaging studies support an association between obesity and hypothalamic gliosis (Schur et al., 2016; Sewaybricker et al., 2023; Rosenbaum et al., 2022). However, there is currently very little direct evidence of astrocytic dysfunction in human cardiovascular regulatory regions like the RVLM, PVN or NTS. Therefore, human induced pluripotent stem cell-derived astrocytes, postmortem single-cell and spatial transcriptomic studies, advanced molecular imaging and three-dimensional neural culture systems may aid in bridging the gap between experimental models and human OIH.

Finally, the therapeutic potential of targeting astrocytes in OIH remains largely unexplored. Although experimental restoration of EAAT2 can attenuate astrocyte-mediated neuronal hyperexcitability in cardiovascular autonomic regions (Danbolt, 2001), its therapeutic efficacy specifically in OIH remains to be established. Future studies should determine whether astrocyte-specific enhancement of EAAT2 or targeting upstream mechanisms such as oxidative stress, NF-κβ mediated inflammation, and metabolic dysfunction can reduce sympathetic activity and BP during obesity (Douglass et al., 2017; Balasubramanian et al., 2019). Overall, future studies should identify the astrocyte populations and molecular pathways that directly contribute to sympathoexcitation and hypertension during obesity. Integrating cell-specific approaches with cardiovascular telemetry, sympathetic nerve recordings, spatial transcriptomics, and multi-omics analyses will be important for establishing causal astrocytic mechanisms and identifying potential therapeutic targets for OIH.

## 8 Conclusion

Obesity-induced hypertension is a complex multifactorial disorder resulting from the integration of metabolic, renal, vascular, inflammatory, and neurogenic mechanisms. Increasing evidence suggests that dysregulation of central autonomic pathways contributes to sustained sympathetic overactivity and increased arterial pressure in obesity. Astrocytes have emerged as active regulators of cardiovascular function rather than merely supportive cells within these mechanistic pathways. Within hypothalamic and brainstem autonomic nuclei, astrocytes can influence neuronal excitability through the regulation of glutamate homeostasis, neuroinflammation, oxidative stress, neurovascular coupling, and blood–brain barrier integrity.

Obesity-associated inflammatory and metabolic signals can disrupt these homeostatic functions by increasing reactive astrogliosis and impaired glutamate clearance, thereby contributing to sympathoexcitation within autonomic regulatory circuits. Disruption of astrocytic EAAT-mediated glutamate homeostasis may provide a mechanistic link between astrocyte dysfunction, neuronal hyperexcitability, and sustained sympathoexcitation. However, despite growing experimental evidence, the causal relationships among astrocytic dysfunction, sympathetic activation, and the development of OIH remain incompletely established.

Future studies are needed to define the region-specific and causal roles of astrocytes in OIH and to determine how these mechanisms change during disease progression. Approaches that restore glutamate homeostasis, reduce maladaptive neuroinflammation and oxidative stress, and preserve astrocyte-neuron communication could complement traditional BP management strategies. Therefore, a better understanding of the role of astrocytes in autonomic regulation of BP may help identify novel therapeutic interventions for the treatment and prevention of obesity-induced hypertension.
